# Bone phenotyping of murine hemochromatosis models with deficiencies of Hjv, Alk2, or Alk3: The influence of sex and the bone compartment

**DOI:** 10.1096/fj.202401015R

**Published:** 2024-11-15

**Authors:** Deniz Y. Dogan, Isabelle Hornung, Mariateresa Pettinato, Alessia Pagani, Ulrike Baschant, Guiscard Seebohm, Lorenz C. Hofbauer, Laura Silvestri, Martina Rauner, Andrea U. Steinbicker

**Affiliations:** ^1^ Department of Anesthesia, Intensive Care and Pain Medicine Goethe University Frankfurt Frankfurt Germany; ^2^ Regulation of Iron Metabolism Unit, Division of Genetics and Cell Biology IRCCS San Raffaele Scientific Institute Milan Italy; ^3^ Department of Medicine III & Center for Healthy Aging, Medical Faculty and University Hospital Carl Gustav Carus Dresden University of Technology Dresden Germany; ^4^ IfGH– Cellular Electrophysiology, Department of Cardiology and Angiology University Hospital of Münster Münster Germany; ^5^ School of Medicine Vita‐Salute San Raffaele University Milan Italy; ^6^ Present address: Faculty of Medicine and University Hospital of Cologne, Department of Anaesthesiology and Intensive Care Medicine University of Cologne Cologne Germany

## Abstract

Osteopenia is frequently observed in patients with iron overload, especially in those with *HFE*‐dependent hereditary hemochromatosis (HH). Interestingly, not all mouse models of HH show bone loss, suggesting that iron overload alone may not suffice to induce bone loss. In this study, the bone phenotypes of *Hjv* ^*−/−*^ and hepatocyte‐specific *Alk2*‐ and *Alk3*‐deficient mice as additional mouse models of HH were investigated to further clarify, how high iron levels lead to bone loss and which signaling mechanisms are operational. Neither male nor female 12‐week‐old *Hjv* ^*−/−*^ mice had an altered trabecular or cortical bone mass or bone turnover, despite severe iron overload. Male 12‐month‐old *Hjv* ^*−/−*^ mice even presented with a higher femoral trabecular bone volume compared to wildtype mice. Similarly, female mice with hepatocyte‐specific *Alk2* or *Alk3* deficiency did not show an altered bone phenotype at 3, 6, and 12 months of age. Male hepatocyte‐specific *Alk3*‐deficient mice also had a normal trabecular bone mass at all ages analyzed, despite showing increased bone resorption and decreased bone formation parameters. Interestingly, hepatocyte‐specific *Alk2*‐deficient mice showed reduced femoral trabecular bone at 6 months of age due to suppressed bone formation. Cortical thickness at the femur was reduced in both, 6‐month‐old male hepatocyte‐specific *Alk2*‐ and *Alk3*‐deficient mice. Raising hepatocyte‐specific *Alk2*‐deficient male mice on an iron‐deficient diet rescued the bone phenotype. Taken together, despite iron overload, trabecular bone microarchitecture was not altered in mice deficient of *Hjv or Alk3*. Only male hepatocyte‐specific *Alk2*‐deficient mice showed site‐specific lower trabecular and cortical bone mass at the femur, which was dependent on iron. Thus, bone loss does not correlate with the extent of iron overload in these mouse models, but may relate to the amount of iron‐loaded macrophages, as precursors of osteoclasts, in the bone marrow.

## Graphical abstract

Iron overload is most severe in Hjv−/− and Alk3fl/fl; Alb‐cre mice, followed by Alk2fl/fl; Alb‐Cre mice and WT mice. Severe hepcidin suppression in Hjv−/− and Alk3fl/fl; Alb‐cre mice leads to lower numbers of iron‐loaded macrophages in the bone marrow. Bone loss is largest in Alk2fl/fl; Alb‐Cre mice, which have the weakest hepcidin suppression and highest number of iron‐loaded cells in the bone marrow.

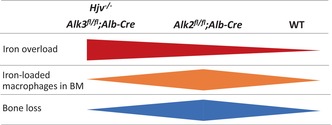

**Abbreviations**
Alk2: BMP type I receptor (Acvr1)
Alk3: BMP type I receptor (Bmpr1a)
ANOVA: analysis of variance
BFR/BS: bone formation rate/bone surface
BMP: bone morphogenetic protein
BV/TV: bone volume/total volume
CTX: cross‐linked C‐telopeptide of type I collagen
Hamp: Hepcidin
HFE: High Fe
HJV: Hemojuvelin
kVp: kilo electron voltage
MAR: mineral apposition rate
MS/BS: mineralizing surface/bone surface
µM: micro molar
ms: milliseconds
P1NP: pro‐collagen type I amino‐terminal propeptide
PPM: Parts per million
SD: standard deviation
TRAP5B: tartrate resistant acid phosphatase 5b
TFR2: tranferrin receptor 2

## 1 INTRODUCTION

Iron is essential for several physiological processes. As such, well‐balanced iron levels are indispensable for health and both, iron deficiency as well as iron overload result in major health problems. While iron deficiency causes anemia, weakness, and shortness of breath, iron overload can lead to failure of the heart, pancreas, and liver due to the accumulation of iron into these organs.^1^, ^2^ In addition to cardiomyopathy, diabetes, and liver failure, osteoporosis and an increased rate of fractures have also been observed in patients with hemochromatosis.^3^, ^4^, ^5^, ^6^, ^7^, ^8^, ^9^ In *HFE*‐dependent hereditary hemochromatosis (HH), osteoporosis is present in up to 34% of the patients,^10^, ^11^, ^12^ with men being more susceptible to an increased risk for osteoporosis.^13^ Besides osteoporosis, also other skeletal manifestations such as osteonecrosis and bone marrow edema have been reported in patients with *HFE*‐HH.^14^ Although the underlying disease mechanisms are not fully understood, similar to other hemochromatosis‐related pathologies (e.g., liver disease, cardiomyopathy), the occurance of osteoporosis is associated with the severity of iron overload.^10^, ^15^, ^16^, ^17^, ^18^, ^19^, ^20^

Systemic iron homeostasis is mainly controlled by the hepatic hormone hepcidin, which regulates iron availability in the blood and thus iron distribution in the body by blocking the sole iron exporter ferroportin. Several other iron regulatory proteins have been characterized, with many of them being involved in the regulation of the signaling pathway involved in hepcidin expression, the bone morphogenetic protein‐son of mother against decapentaplegic homolog (BMP‐SMAD) signaling pathway. Deficiency of these iron regulators leads to abnormally low hepcidin levels and iron overload of various grades. As reviewed by Bartnikas et al., hepatocyte‐specific deficiency of the BMP type I receptor *Alk2* and *Hfe* in mice leads to moderate iron overload compared to wildtype littermates,^21^ while in contrast, global or hepatocyte‐specific deficiency of *Alk3*, *Hjv* (hemojuvelin), and *Hamp* (hepcidin) are characterized by severe iron overload with up to five‐fold elevated liver iron content compared to controls due to severe depletion of hepcidin levels.^21^, ^22^, ^23^, ^24^, ^25^ Of note, also deficiency of the BMP ligands *Bmp2* and *Bmp6* in liver sinusoidal endothelial cells leads to severe iron overload.

In an effort to better understand the underlying mechanisms of iron‐induced bone loss, various mouse models of hemochromatosis have been employed. Initial studies of *Hfe* ^*−/−*^ mice showed low bone mass associated with reduced bone formation and increased numbers of osteoclasts.^26^, ^27^ However, no bone phenotype was observed in subsequent studies using *Hfe* ^*−/−*^, liver‐specific *Hfe* as well as osteoblast‐ and osteoclast‐specific *Hfe* knockout mice of various ages.^28^, ^29^ Interestingly, the lack of bone loss is not only evident in *Hfe*‐deficient mice, but also in mice lacking the iron sensor transferrin receptor 2 (Tfr2). Unexpectedly, these mice display a high bone mass phenotype that is independent of iron overload, being present also in animals lacking *Tfr2* in osteoblasts, demonstrating a cell type‐specific role of TFR2.^30^ Deficiency of hepcidin or the use of hepcidin‐resistant ferroportin transgenic mice was shown to cause inhibition of bone formation and low bone mass in mice and zebrafish.^31^, ^32^, ^33^, ^34^ At the same time, mice overexpressing hepcidin in hepatocytes or osteoblasts also lead to bone loss due to increased osteoclast activity.^35^ Finally, interference with the BMP signaling pathway led to various bone outcomes: blockade of BMP signaling with dorsomorphin resulted in iron overload and suppression of bone formation.^36^ Global deficiency of *Bmp6* resulted either in low^37^ or normal^38^ trabecular bone mass, and finally, deficiency of *Bmp2* in liver sinusoidal endothelial cells showed no gross skeletal abnormalities.^39^ Overall, these studies do not yet provide a clear picture about the underlying mechanisms of iron‐induced bone loss.

Thus, in this study, we aimed to investigate the bone phenotype of three additional mouse models of genetic iron overload: *Hjv* ^*−/−*^ mice, which are amongst the most severely iron overloaded mice, as well as mice with a hepatocyte‐specific deficiency of *Alk2* (moderate iron overload) and of *Alk3* (severe iron overload comparable to *Hjv* ^*−/−*^ mice).

## 2 MATERIALS AND METHODS

### 2.1 Mouse cohorts

*Hepatocyte‐specific Alk2‐* or *Alk3‐deficient mice:* Mouse breeding and animal procedures were approved and conducted in compliance with the guidelines of the institutional animal care committee and the Regierungspräsidium Darmstadt (permit number FK/2028). Male and female hepatocyte‐specific *Alk2* (*Alk2* ^*fl/fl*^ *; Alb‐Cre*) or *Alk3* (*Alk3* ^*fl/fl*^ *; Alb‐Cre*) conditional knockout mice and *Cre‐* littermates on a C57BL/6J background were fed a standard rodent diet (188 ppm iron) with water ad libitum.^40^ A cohort of male hepatocyte‐specific *Alk2* (*Alk2* ^*fl/fl*^ *; Alb‐Cre*) conditional knockout mice also received an iron‐deficient diet (<10 ppm iron, Ssniff) from weaning (around 21 days old) until sacrifice (6 months old). Mice were exposed to a 12 h light/dark cycle and an air‐conditioned SPF room at 23°C (no specific pathogen‐free room). Enrichment was provided in the form of cardboard houses and bedding material. Mice were euthanized at the age of 12 weeks, at 6 or 12 months under deep anesthesia and blood, organs and bones were collected for further analysis.

*Hjv global knockout mice:* Male and female, 12‐week‐old and 12‐month‐old *Hjv* ^*−/−*^ mice and WT mice on an inbred 129S6/SvEvTac background were used and housed under a standard 12‐hour light/dark cycle with water and chow ad libitum in a pathogen‐free animal facility of the San Raffaele Scientific Institute in accordance with the European Union guidelines (IACUC n° 1026).^41^ Mice were fed a standard rodent diet (Safe diets, Essingen, Germany, irradiated complete universal vegetal diet, 150 SP‐25 with 188 ppm iron) with water ad libitum and analyzed at 12 weeks of age.

### 2.2 Iron measurements

Non‐heme iron content in the livers was measured using the bathophenanthroline colorimetric method as previously described.^30^ In brief, 100 mg of liver tissue was dried for 3 days at 37°C, and afterwards the samples were digested, followed by the reaction with 0.01% bathophenanthrolinedisulfonic acid. Values were recorded spectrophotometrically at 535/540 nm. Non‐heme iron content is reported as μg iron/g dry tissue weight.

Further, iron and transferrin saturation were measured in the serum using the total iron binding capacity kit from Randox as previously described.^30^

### 2.3 μCT analysis of bone microarchitecture

The distal femur and the fourth lumbar vertebrae were excised, and their bone microarchitecture was measured using a vivaCT40 (ScancoMedical, Switzerland). Images were taken at an isotropic voxel‐size of 10.5 μm with X‐ray energy of 70 kVp/114 μA and 200 ms of integration time. For femora, half the femur was scanned, and 100 slices below the growth plate of the distal femur was evaluated for trabecular bone, and 150 slices in the mid‐diaphysis were evaluated for cortical bone. For the vertebral bone, the entire 4th lumbar vertebrae was measured, and 100 slices in the middle of the bone were measured. Trabecular and cortical bone parameters were assessed using standard protocols from Scanco Medical. μCT parameters are reported according to international guidelines.

### 2.4 Bone histomorphometry

Hepatocyte‐specific *Alk2;Alb‐Cre*‐ and *Alk3‐Alb‐Cre*‐deficient mice, *Hjv* ^*−/−*^ mice, and control animals received two intraperitoneal injections with 20 mg/kg calcein (Sigma) five and 2 days before sacrifice. For dynamic bone histomorphometry, the third and fourth lumbar vertebrae were fixed in 4% PBS‐buffered paraformaldehyde and dehydrated in an ascending ethanol series. Subsequently, bones were embedded in methacrylate and cut into 7 μm sections to assess the fluorescent calcein labels. Sections were analyzed using fluorescence microscopy to determine the mineralized surface/bone surface (MS/BS), the mineral apposition rate (MAR), and the bone formation rate/bone surface (BFR/BS).

To determine numbers of osteoclasts, the fifth lumbar vertebra was decalcified for 1 week using Osteosoft (Merck), dehydrated, and embedded into paraffin. Tartrate‐resistant acid phosphatase (TRAP) staining was used to identify osteoclasts. These sections were also used to quantify the number of osteoblasts based on their location and morphology. Bone sections were analyzed using the Osteomeasure software (Osteometrics, USA).

Iron was stained on paraffin‐embedded bone sections using Perls' Prussian blue staining.^42^

### 2.5 Serum analysis of bone turnover markers

Concentrations of pro‐collagen type I amino‐terminal propeptide (P1NP) and tartrate resistant acid phosphatase 5b (TRAP5b) were quantified in the serum using enzyme‐linked immunosorbent assays from Immundiagnostik, Bensheim, Germany.

### 2.6 Statistical analysis

Data are presented as mean ± standard deviation (SD). Two‐way ANOVA for comparison of age and genotype or a two‐sided unpaired Student's *t*‐tests were used to compare two groups. Calculations were performed using GraphPad Prism 8 (GraphPad Software Inc., San Diego, CA, USA). *p*‐values <.05 were considered statistically significant.

## 3 RESULTS

### 3.1 Hjv knockout mice do not display bone loss despite iron overload

**FIGURE 1:**
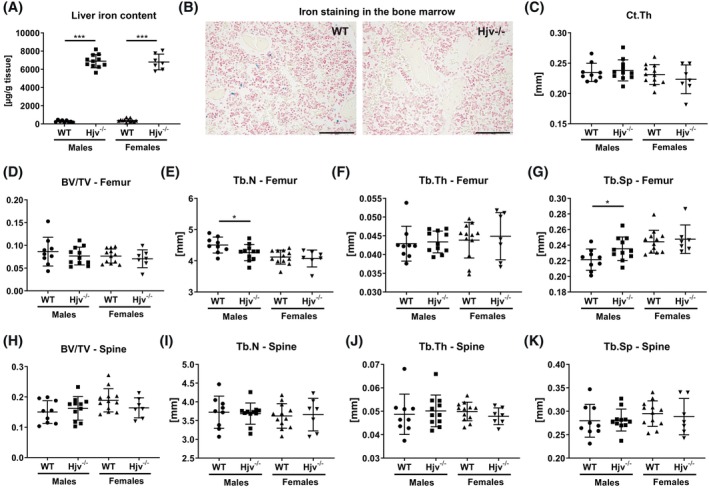
Bone phenotype of *Hjv* ^*−/−*^ mice. Twelve‐week old male and female Hjv^−/−^ and wildtype mice were analyzed. (A) Liver iron content, (B) iron staining of the bone/bone marrow, (C) cortical thickness (Ct.Th) of the femoral mid‐diaphysis, (D) trabecular bone volume/tissue volume (BV/TV), (E) trabecular number (Tb.N), (F) trabecular thickness (Tb.Th), (G) trabecular separation (Tb.Sp) of the distal femur. (H) Trabecular bone volume/tissue volume (BV/TV), (I) trabecular number (Tb.N), (J) trabecular thickness (Tb.Th), (K) trabecular separation (Tb.Sp) of the fifth lumbar vertebra. Individual dots represent individual mice. Mean and SD are indicated as horizontal lines. A two‐sided *t*‐test was used for statistical analysis. *p < .05; ***p < .001.

To investigate whether *Hjv* ^*−/−*^ mice, which are severely iron overloaded, exhibit bone loss, we conducted a microarchitectural, histological, and serological characterization of bone. Analysis of the liver iron content confirmed that both, 12‐week‐old male and female *Hjv* ^*−/−*^ mice had exceedingly high levels of iron stored in the liver (Figure 1A). Perl's Prussian Blue staining revealed iron‐loaded macrophages in the bone marrow of male wildtype mice, but much fewer in male *Hjv* ^*−/−*^ mice (wildtype: 48 ± 57/mm^2^, *Hjv* ^*−/−*^: 2.50 ± 1.75/mm^2^, *p* = .0042) (Figure 1B), as expected with hepcidin being strongly reduced and ferroportin stabilized in *Hjv* ^*−/−*^ mice. No staining or iron was found adjacent to the bone surface (Figure 1B). Neither male nor female *Hjv* ^*−/−*^ mice displayed alterations in the femoral cortical thickness (Figure 1C). At the trabecular bone compartment, no changes in the bone volume fraction were observed in the distal femur (Figure 1D). However, trabecular number was decreased in male *Hjv* ^*−/−*^ mice, whereas trabecular separation was increased and trabecular thickness was unchanged compared to wild‐type littermates (Figure 1E–G). No changes in trabecular bone structure were observed in the fourth vertebral body (Figure 1H–K).

**FIGURE 2:**
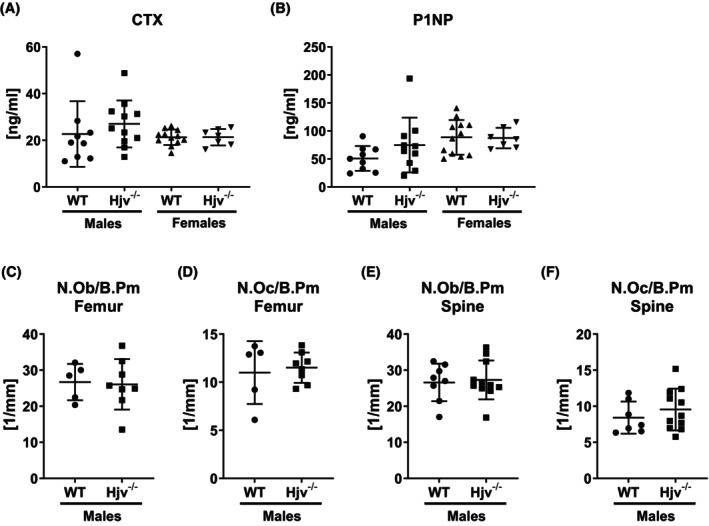
Bone turnover in *Hjv* ^*−/−*^ mice. (A) Serum C‐terminal telopeptide of type I collagen (CTX) levels and (B) serum procollagen type I N‐terminal peptide (P1NP) levels in male and female 12‐week‐old Hjv^−/−^ and wildtype littermate controls. (C–F) The number of osteoblasts per bone perimeter (N.Ob/B.Pm) and the number of osteoclasts per bone perimeter (N.Oc/B.Pm) was assessed at the fifth lumbar vertebra and distal femur of male mice. Individual dots represent individual mice. Mean and SD are indicated as horizontal lines. A two‐sided *t*‐test was used for statistical analysis to compare genotypes of the same sex.

In line with the bone microarchitectural data, serum levels of the bone turnover markers CTX (indicating bone resorption) and P1NP (indicating bone formation) in males and females, as well as numbers of osteoclasts and osteoblasts at the femur and spine as assessed using histology in male bones, were not different between the genotypes (Figure 2A–F).

To investigate if iron overload induces bone loss over time, 12‐month‐old *Hjv* ^*−/−*^ mice were analyzed as well. As shown in Figure S1, despite increased liver iron levels in male and female *Hjv* ^*−/−*^ mice, they did not show trabecular or cortical bone loss. In fact, males even presented with a higher trabecular bone volume at the distal femur with no significant alterations in trabecular parameters, compared to age‐matched WT controls (Figure S1). The fourth lumbar vertebrae did not show major alterations (Figure S1D). The serum bone turnover makers CTX and P1NP were unchanged in aged male and female *Hjv* ^*−/−*^ mice (Figure S2A,B). Likewise, osteoclast parameters at the femur and spine were normal in *Hjv* ^*−/−*^ mice; however, the bone formation rate at the femur was increased in *Hjv* ^*−/−*^ mice, suggesting that increased bone formation may be causing the higher femoral bone mass of *Hjv* ^*−/−*^ mice (Figure S2C–F). Taken together, despite severe iron overload, *Hjv* ^*−/−*^ mice do not have a low bone mass phenotype.

### 3.2 Hepatocyte‐specific Alk3‐deficient male mice present cortical bone loss associated with higher number of osteoclasts and lower bone formation

**FIGURE 3:**
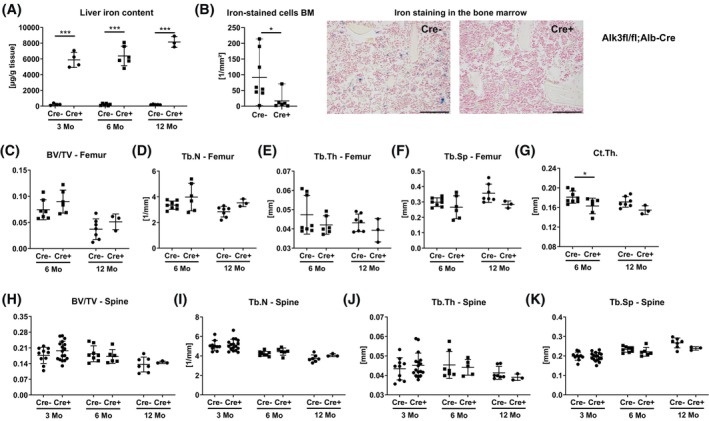
Bone microarchitecture of *Alk3* ^*fl/fl*^ *; Alb‐Cre* mice assessed with μCT. Male and female *Alk3* ^*fl/fl*^ *; Alb‐Cre* positive and negative mice of different ages were analyzed. (A) Liver iron content and (B) iron‐stained cells in the bone marrow with representative images. Scale bar: 100 μm. (C) Trabecular bone volume/tissue volume (BV/TV), (D–F) trabecular number, thickness and separation at the distal femur, and (G) cortical thickenss at the femoral midshaft, (H) trabecular bone volume, (I–K) trabecular number, thickness and separation at the fourth vertebral body. Individual dots represent individual mice. Mean and SD are indicated as horizontal lines. A two‐sided *t*‐test was used for statistical analysis to compare genotypes of the same age. *p < .05, ***p < .001.

Next, we examined the bone phenotype in mice with hepatocyte‐specific *Alk3* deficiency, which exhibit iron‐overload comparable to the genetic hemochromatosis model *Hjv* ^*−/−*^ mice. As previously published, the liver iron content was higher amongst the male *Alk3* ^*fl/fl*^ *; Alb‐Cre* mice across all ages compared to *Alk3* ^*fl/fl*^ controls (Figure 3A). Moreover, serum iron levels (incl. ferrous and ferric iron) and transferrin saturation were higher in male *Alk3* ^*fl/fl*^ *; Alb‐Cre* mice at 6 months of age (Table 1). Similar to *Hjv* ^*−/−*^ mice, *Alk3* ^*fl/fl*^ *; Alb‐Cre* mice showed a reduced number of iron‐loaded macrophages in the bone marrow (Figure 3B). Trabecular bone volume and structural parameters at the lumbar spine and the distal femur were not changed in male *Alk3* ^*fl/fl*^ *; Alb‐Cre* mice at any age investigated, although the femoral bone volume showed tendencies to be increased (Figure 3C–F, H–K). Interestingly, cortical thickness was decreased in *Alk3* ^*fl/fl*^ *; Alb‐Cre* mice at 6 months of age and tended to be decreased at 12 months of age (Figure 3G).

**TABLE 1:** Serum iron levels and transferrin saturation in male 12‐week‐old Hjv^−/−^ mice and male 6‐month‐old hepatocyte‐specific Alk2‐ and Alk3‐deficient mice.

	WT	*Hjv* ^*−/−*^	*Alk3* ^*fl/fl*^	*Alk3* ^*fl/fl*^ *; Alb‐Cre*	*Alk2* ^*fl/fl*^	*Alk2* ^*fl/fl*^ *; Alb‐Cre*
Serum total iron (μM)	79.9 ± 17.7	130.0 ± 42.9**	50.8 ± 16.5	84.0 ± 9.5*	52.6 ± 8.4	59.9 ± 8.8
Serum ferrous iron (μM)	35.8 ± 9.4	51.4 ± 26.9	24.4 ± 2.7	43.5 ± 7.6**	21.7 ± 1.1	29.8 ± 3.4**
Serum ferric iron (μM)	44.2 ± 10.2	78.6 ± 17.9***	26.4 ± 18.2	40.5 ± 13.5*	30.9 ± 7.8	30.2 ± 6.9
Transferrin saturation (%)	n.a.	n.a.	33.3 ± 8.2	84.8 ± 12.5***	39.2 ± 12.3	64.4 ± 19.4*

* *p* < .05;

** *p* < .01;

*** *p* < .001.

**FIGURE 4:**
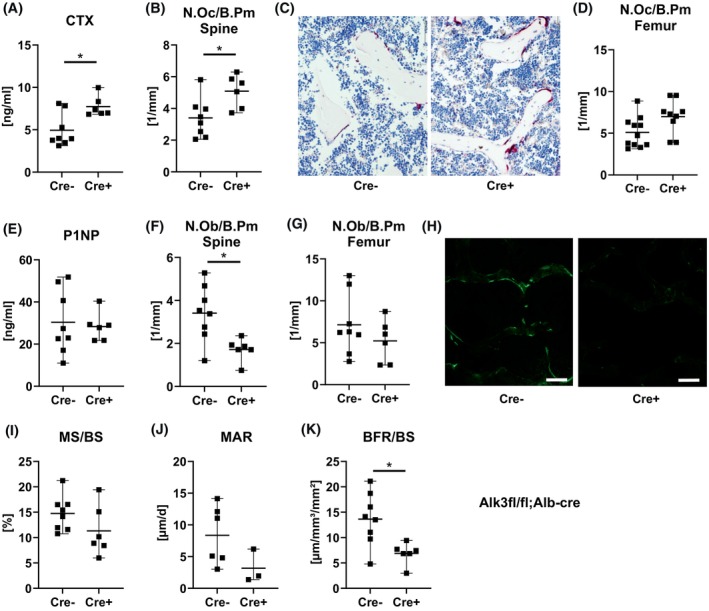
Histological and serological analysis of bone turnover of male *Alk3* ^*fl/fl*^ *; Alb‐Cre* mice. Bone turnover parameters were analyzed in the serum and at the lumbar vertebrae and femur of male 24‐week‐old *Alk3* ^*fl/fl*^ *; Alb‐Cre* mice. (A) Serum C‐terminal telopeptide of type I collagen (CTX) levels, (B) number of osteoclasts per bone perimeter (N.Oc/B.Pm) in the fourth lumbar vertebral body, (C) representative images of TRAP‐stained vertebral sections, scale bar: 100 μm, and (D) number of osteoclasts per bone perimeter in the femoral bone. (E) Serum procollagen type I N‐terminal peptide (P1NP) levels, (F–G) number of osteoblasts per bone perimeter (N.Ob/B.Pm) in spine and femur, and (H) representative images of calcein vertebral sections, scale bar: 100 μm. (I) Mineralizing surface per bone surface (MS/BS), (J) mineral apposition rate (MAR), and (K) bone formation rate per bone surface (BFR/BS) at the fourth vertebral body. Individual dots represent individual mice. Mean and SD are indicated as horizontal lines. A two‐sided *t*‐test was used for statistical analysis. *p < .05.

We next performed serological and histological bone analyses of the bones of 6‐month‐old *Alk3* ^*fl/fl*^ *; Alb‐Cre* mice. Despite showing no alterations in bone microarchitecture, the bone resorption marker CTX and the number of osteoclasts in the femur and spine were both increased by 35–45% in *Alk3* ^*fl/fl*^ *; Alb‐Cre* mice (Figure 4A–D). While the bone formation marker P1NP was not changed in *Alk3* ^*fl/fl*^ *; Alb‐Cre* mice, the number of osteoblasts at the spine (but not the femur) decreased by half in *Alk3* ^*fl/fl*^ *; Alb‐Cre* mice (Figure 4E–G). In line with these findings, dynamic bone histomorphometry showed fewer calcein labels in the fourth vertebral body of *Alk3* ^*fl/fl*^ *; Alb‐Cre* mice compared to controls (Figure 4H). Quantification of the labels showed that the mineralizing surface per bone surface and the mineral apposition rate tended to be decreased, resulting in a significantly dereased bone formation rate (Figure 4I–K).

Taken together, hepatocyte‐specific deficiency of *Alk3* does not only lead to iron overload in blood and organs, but also altered bone remodeling. Interestingly, this did not lead to changes in trabecular bone microarchitecture, but to femoral cortical bone loss at 6 months of age.

### 3.3 Hepatocyte‐specific Alk2‐deficient male mice show femoral bone loss

**FIGURE 5:**
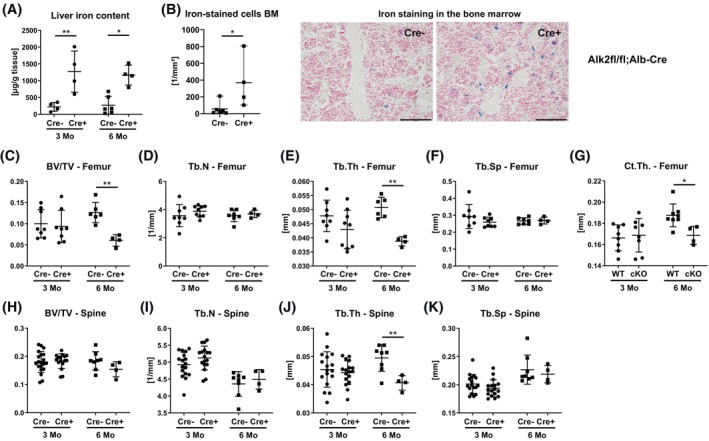
Bone microarchitecture of *Alk2; Alb‐cre* cKO mice assessed with μCT. Male *Alk2* ^*fl/fl*^ *controls* and *Alk2* ^*fl/fl*^ *; Alb‐Cre* mice of different ages were analyzed. (A) Liver iron content and (B) iron‐stained cells in the bone marrow with representative images. Scale bar: 100 μm. (C) Trabecular bone volume/tissue volume (BV/TV), (D–F) trabecular number, thickness and separation at the distal femur, and (G) cortical thickness at the femoral midshaft, (H) trabecular bone volume, (I–K) trabecular number, thickness and separation at the fourth vertebral body. Indiviual dots represent individual mice. Mean and SD are indicated as horizontal lines. A two‐sided *t*‐test was used for statistical analysis to compare genotypes of the same age and sex. *p < .05, **p < .01.

We next analyzed the bone phenotype of *Alk2* ^*fl/fl*^ *; Alb‐Cre* mice, which have a milder iron overload phenotype compared to *Hjv* ^*−/−*^ and *Alk3* ^*fl/fl*^ *; Alb‐Cre* mice, as displayed by higher liver iron levels (Figure 5A) as well as higher serum iron and transferrin saturation levels (Table 1) as compared to their *Alk2* ^*fl/fl*^ littermate controls. In contrast to *Hjv* ^*−/−*^ and *Alk3* ^*fl/fl*^ *; Alb‐Cre* mice, *Alk2* ^*fl/fl*^ *; Alb‐Cre* mice showed a higher number of iron‐stained cells in the bone marrow (Figure 5B). Male 6‐month‐old *Alk2* ^*fl/fl*^ *; Alb‐Cre* mice showed a 50% reduction in trabecular bone volume, along with a reduction in trabecular thickness, while trabecular number and separation were unchanged (Figure 5C–F). Cortical thickness at the femoral midshaft was reduced by 10% (Figure 5G). No changes in femoral bone microarchitecture were observed in 3‐month‐old male *Alk2* ^*fl/fl*^ *; Alb‐Cre* mice (Figure 5C–G). Besides reduced trabecular thickness, no alterations in trabecular bone structure were observed in the fourth lumbar vertebrae of 3‐ or 6‐month‐old *Alk2* ^*fl/fl*^ *; Alb‐Cre* male mice (Figure 5H–K). Female *Alk2* ^*fl/fl*^ *; Alb‐Cre* mice showed no changes in their trabecular or cortical bone mass, neither at the femur nor at the spine (Figure S3).

**FIGURE 6:**
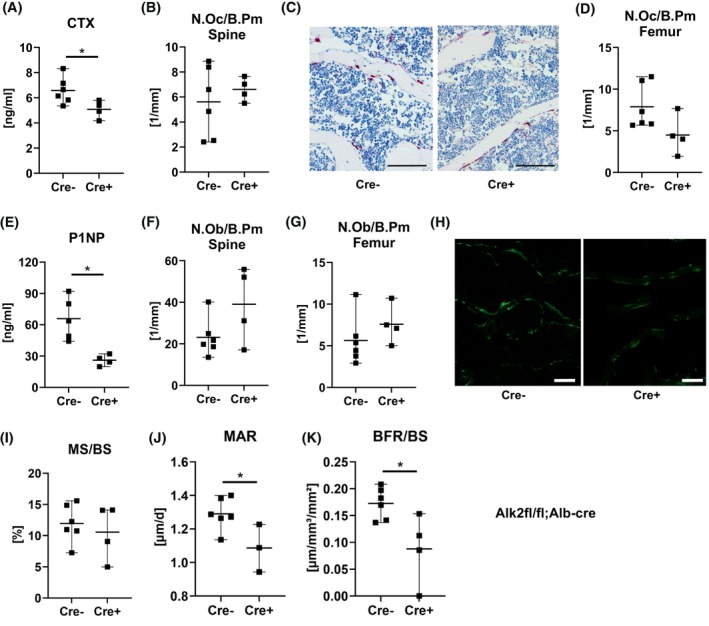
Histological and serological analysis of bone turnover of male *Alk2* ^*fl/fl*^ *; Alb‐Cre* mice. Bone turnover parameters were analyzed in the serum and at the lumbar vertebrae of male 24‐week‐old *Alk2* ^*fl/fl*^ *; Alb‐Cre* mice. (A) Serum C‐terminal telopeptide of type I collagen (CTX) levels, (B) number of osteoclasts per bone perimeter (N.Oc/B.Pm) in the fourth lumbar vertebral body, (C) representative images of TRAP‐stained vertebral sections, scale bar: 100 μm, and (D) number of osteoclasts per bone perimeter in the femoral bone. (E) Serum procollagen type I N‐terminal peptide (P1NP) levels, (F and G) number of osteoblasts per bone perimeter (N.Ob/B.Pm) in spine and femur, and (H) representative images of calcein vertebral sections, scale bar: 100 μm. (I) Mineralizing surface per bone surface (MS/BS), (J) mineral apposition rate (MAR), and (K) bone formation rate per bone surface (BFR/BS) at the fourth vertebral body. Individual dots represent individual mice. Mean and SD are indicated as horizontal lines. A two‐sided *t*‐test was used for statistical analysis. *p < .05.

At a cellular level, male *Alk2* ^*fl/fl*^ *; Alb‐Cre* mice showed lower serum CTX levels, while no changes in osteoclast parameters were found histologically at the femur or spine (Figure 6A–D). Likewise, the number of osteoblasts and the mineralizing surface were not different between the genotypes at the spine or femur, but serum levels of P1NP, the mineral apposition rate, and the bone formation rate were significantly decreased in *Alk2* ^*fl/fl*^ *; Alb‐Cre* mice (Figure 6E–K).

Taken together, *Alk2* ^*fl/fl*^ *; Alb‐Cre* male mice show reduced trabecular bone parameters at the age of 6 months, likely due to a suppression of bone formation.

### 3.4 Low iron diet rescues femoral bone loss in hepatocyte‐specific Alk2‐deficient mice

**FIGURE 7:**
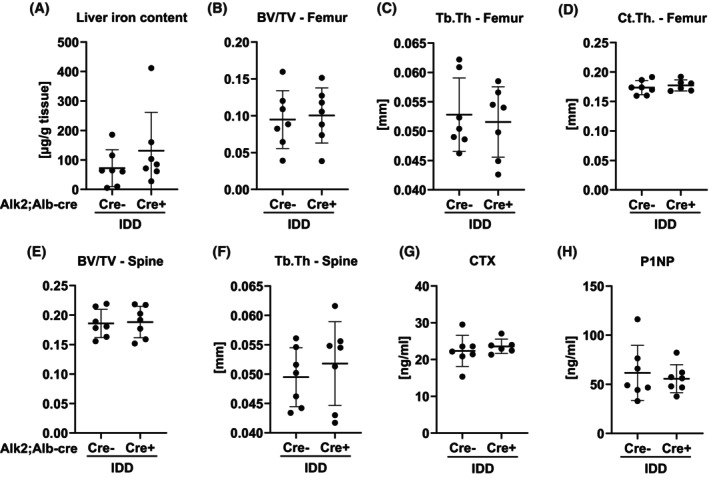
Iron‐deficient diet rescues the femoral bone phenotype of *Alk2* ^*fl/fl*^ *; Alb‐Cre* mice. Male *Alk2* ^*fl/fl*^ *controls* and *Alk2* ^*fl/fl*^ *; Alb‐Cre* mice were put on an iron‐deficient diet (IDD) after weaning. At the age of 24 weeks, mice were analyzed regarding their liver iron content (A) and bone phenotype (B–H). (B) Trabecular bone volume/tissue volume (BV/TV) and (C) trabecular thickness at the distal femur. (D) Cortical thickness (Ct.Th) of the mid‐diaphysis. (E) Trabecular bone volume/tissue volume (BV/TV) and (F) trabecular thickness at the fourth lumbar vertebrae. (G) CTX and (H) P1NP serum levels. Individual dots represent individual mice. Mean and SD are indicated as horizontal lines. A two‐sided *t*‐test was used for statistical analysis.

Given the decreased femoral trabecular volume and cortical thickness and the higher number of iron‐stained cells in the bone marrow of 6‐month‐old *Alk2* ^*fl/fl*^ *; Alb‐Cre*‐deficient male mice, we wondered whether femoral bone loss was due to their higher systemic iron levels. Therefore, we fed *Alk2* ^*fl/fl*^ *; Alb‐Cre* male mice a low iron diet after weaning until 6 months of age. This diet prevented the increase in liver iron levels (Figure 7A) as well as the femoral trabecular and cortical bone loss in *Alk2* ^*fl/fl*^ *; Alb‐Cre* mice (Figure 7B–D). Trabecular bone volume and trabecular thickness of the fourth lumbar vertebrae also remained unchanged in *Alk2* ^*fl/fl*^ *; Alb‐Cre* mice on the low iron diet (Figure 7E,F). This is consistent with the measurement of bone turnover markers, which showed no difference between genotypes (Figure 7G,H). Thus, the iron‐deficient diet normalized the femoral bone phenotype of the *Alk2* ^*fl/fl*^ *; Alb‐Cre* mice, suggesting that the higher iron load drives femoral bone loss.

## 4 DISCUSSION

Bone loss has been reported frequently in HH patients as well as in some mouse models of iron overload, including *Hfe* ^*−/−*^ mice, *Bmp6* ^*−/−*^ mice, and hepcidin‐deficient mice,^3^, ^10^, ^27^, ^32^, ^34^, ^37^, ^43^ although with controvertial results. In contrast, *Tfr2* ^*−/−*^ mice and mice with a deletion of *Bmp2* in liver sinusoidal endothelial cells showed no obvious bone loss,^28^, ^29^, ^30^, ^39^, ^44^ despite hepcidin deficiency and body iron overload.

To better understand under which circumstances iron overload leads to bone loss, we explored the bone phenotypes of *Hjv* ^*−/−*^ as well as hepatocyte‐specific *Alk2*‐ and *Alk3*‐deficient mice as additional mouse models with HH. While *Hjv* ^*−/−*^ and hepatocyte‐specific *Alk3*‐deficient mice are severely iron overloaded and have almost undetectable hepcidin expression levels in the liver, hepatocyte‐specific *Alk2*‐deficient mice are only moderately iron‐overloaded with lower, but detectable levels of hepcidin mRNA in the liver.^21^, ^41^ At 12 weeks of age, none of the three models showed trabecular bone abnormalties. Suprisingly, hepatocyte‐specific *Alk2*‐deficient mice, despite having the lowest iron load, had the strongest bone phenotype at 6 months of age, showing reduced trabecular and cortical bone volume. Six month old male hepatocyte‐specific *Alk3*‐deficient mice also showed cortical bone loss. At 12 months of age, male *Hjv* ^*−/−*^ and to some extent male hepatocyte‐specific *Alk3*‐deficient mice even showed an increase in trabecular bone mass. These phenomena were present only in male mice, while female mice showed no bone abnormalities at any of the ages examined.

To date, most studies investigating the effects of iron overload on bone only examined trabecular bone parameters, while cortical bone parameters were often neglected. Focusing on trabecular bone, it was very suprising that no bone loss was observed in any of the three models examined here, despite the extent of iron overload. In fact, older *Hjv* ^*−/−*^ and hepatocyte‐specific *Alk3*‐deficient mice even had or tended to have higher trabecular bone mass at the femur, reminiscent of the bone phenotype of *Tfr2* ^*−/−*^ mice, although here, increased bone volume was already present in younger mice and also at the spine.^30^ In all three cases (*Tfr2* ^*−/−*^, *Hjv* ^*−/−*^ and hepatocyte‐specific *Alk3*‐deficient mice), the increase in bone volume was higher in the femur than the vertebral bone, suggesting that potentially a factor in the bone marrow compartment may affect bone turnover in the femoral bone. However, this notion needs to be tested in future studies.

Besides the lack of a major trabecular bone phenotype in our models, it was striking to note that cortical bone loss was detected in male hepatocyte‐specific *Alk2*‐ and *Alk3*‐deficient mice at 6 months of age. Raising the *Alk2* ^*fl/fl*^ *; Alb‐Cre* mice on an iron‐deficient diet showed that the phenotype was iron‐dependent. So far, not too many studies have investigated the cortical bone compartment in hemochromatosis. Previously, studies in 30‐week‐old *Hfe* ^*−/−*^ mice and in humans with *HFE*‐HH showed that the cortical bone compartment was negatively affected, while the trabecular bone compartment was not.^14^, ^29^ Explanations for the differential susceptibility of cortical vs. trabecular bone may stem from a differential expression of iron regulators in trabecular vs. cortical bone, from a different exposure to iron via the bone vasculature, or different machineries for redox detoxifying systems. None of these possibilities have yet been investigated in detail and remain the subject of future investigations. Finally, the cortical bone phenotype was only detected in males, but not in females. However, these sex differences are in line with previous studies that examined iron effects on bone, which show that males are more severely affected by iron overload than females, which may be due to the fact that females are better able to cope with iron fluctuations and that females are less iron overloaded per se.^13^, ^45^

One explanation for the differential cortical bone phenotypes of our models may be the hepatocyte‐specific deletion of *Alk2* and *Alk3* vs. the global deficiency of *Hjv*. As for TFR2, which has been shown to have an osteoblast‐intrinsic role in bone remodeling,^30^ also HJV is expressed in bone cells at low levels^34^ and thus, may participate in the regulation of bone mass. Investigations of iron homeostasis in the liver have revealed that HJV is a BMP co‐receptor, and that HJV signaling is dependent on the BMP type I receptors ALK2 and ALK3.^46^ Neogenin, which is a multifunctional transmembrane receptor belonging to the immunoglobulin superfamily that forms the core of a signal transduction hub for HJV, has also been shown to play a role in endochondral bone formation by promoting lipid raft localization of repulsive guidance molecules and BMP receptors.^47^ Thus, with HJV being a repulsive guidance molecule, further studies should aim at investigating bone cell‐specific *Hjv*‐deficient mice to clarify a potential bone‐intrinsic role of HJV in the regulation of bone remodeling.

Overall, it was surprising that the severely iron overloaded models (*Hjv* ^*−/−*^ and the hepatocyte‐specific *Alk3* deficiency) did not show more pronounced bone loss. Spite the large extent of iron overload in the liver, iron did not accumulate in or along the bone tissue and was in fact depleted in macrophages found in the bone marrow of *Hjv* ^*−/−*^ and *Alk3* ^*fl/fl*^ *; Alb‐Cre* mice, in agreement with the stabilization of ferroportin, highly expressed in macrophages, due to hepcidin deficiency and thus less intracellular iron. This is in line with another previous finding in *Hfe* ^*−/−*^ and hepatocyte‐specific *Hfe*‐deficient mice^29^ and with our own previous observations in *Hfe* ^*−/−*^ mice, which also showed lower numbers of iron‐stained macrophages in the bone marrow and no iron staining of bone tissue.^34^ However, it is in contrast to previous publications showing iron staining along the trabecular bone in *Hfe* ^*−/−*^ mice.^28^, ^37^ Interestingly, these studies also show high iron levels in macrophages, which is rather surprising, given that the low expression of hepcidin in *Hfe* ^*−/−*^ mice should result in higher levels of ferroportin in macrophages. Along those lines, in ferroportin (*Fpn* ^*C326S*^) mutant mice, in which ferroportin is resistant to hepcidin‐mediated degradation, no iron‐stained macrophages were found at all in the bone marrow.^34^ The lack of trabecular bone phenotype in *Hjv* ^*−/−*^ and hepatocyte‐specific *Alk3*‐deficient mice may be related to the actions of hepcidin, or the deficiency here. Previously, it was hypothesized that the lack of hepcidin in HH models leads to increased release of iron from macrophages into the blood stream that thereafter also deposits in bone. In the case of iron dextran injections, hepcidin levels remain high and lead to limited release of iron into the circulation and no bone loss in this particular model of iron dextran injection.^37^ However, an alternative hypothesis may be that the low availability of hepcidin in HH leads to the stabilization of ferroportin on bone cells, allowing them to remove excess intracellular iron, which would otherwise be harmful due to the production of free radicals. This in fact would be supported by the bone phenotypes of hepatocyte‐specific *Tfr2* ^*−/−*^ and *Hfe* ^*−/−*^ mice, which are iron overloaded, but show no bone loss,^29^, ^30^, ^39^ and the hepatocyte‐specific *Alk3*‐deficient mice, which also show no major trabecular bone loss. In contrast, administration of iron to animals would lead to high hepcidin levels, low ferroportin expression on bone cells, and thus, the accumulation of iron and its toxic effects in bone cells and eventually to bone loss. Besides the study of Robin et al. that did not show bone loss after iron dextran injection, several studies have shown bone loss in mice and rats treated with iron dextran, likely due to iron‐induced oxidative stress.^48^, ^49^, ^50^, ^51^ One of the main differences between the studies showing bone loss after iron dextran administration,^48^, ^49^, ^50^, ^51^ and the study by Robin et al. showing no bone loss, is the weekly injection of 100–500 mg/kg iron dextran over 12 weeks compared to a single injection of iron dextran (1000 mg/kg) with analyses 4 months later in the Robin study,^37^ which may lead to a different level of iron overload. However, all studies have in common that they show an increase in the number of iron‐stained cells (macrophages) in the bone marrow, indicating that iron accumulates in macrophages after iron treatment.^37^, ^51^ While no study has investigated the iron load of bone cells in vivo directly, an increased expression of markers of oxidative stress was found in the bone tissue of iron dextran‐treated mice, which may stem from excess intracellular iron levels.^48^ Finally, one argument against our proposed working model is that hepcidin‐deficient mice show bone loss mostly due to reduced bone formation and that hepcidin treatment has been shown to protect against iron‐mediated suppression of bone formation and ovariectomy‐induced bone loss.^32^, ^33^, ^43^, ^52^, ^53^ However, again, opposing results have been published with hepcidin overexpression in hepatocytes or osteoblasts also leading to bone loss due to increased osteoclastogenesis.^35^ Currently, there is a lack of consistency in reporting bone phenotypes in iron overload models, such as e.g., detailed μCT analyses (e.g., analyses of at least two different skeletal sites and the differentiation between cortical and trabecular bone), provision of dynamic histomorphometry parameters and bone turnover markers, as well as bone iron concentrations, so that it remains challenging to produce a unifying working model about the effects of iron on bone at this point.

In summary, our data show that *Hjv* ^*−/−*^ mice with severe iron overload show no bone loss. In contrast, male, but not female, hepatocyte‐specific *Alk2*‐ or *Alk3*‐deficient mice show cortical bone loss with minor alterations in trabecular bone mass. Loss of bone mass in hepatocyte‐specific *Alk2*‐deficient mice is iron‐dependent. Whether the hepcidin deficiency protects trabecular bone from iron toxicity remains to be investigated in future studies.

## AUTHOR CONTRIBUTIONS

Conceptualization: DYD, LS, MR, AUS; Data curation: DYD, IH, LS, UB, GS, MR, AUS; Formal analysis and methodology: DYD, IH, LS, UB, GS, MR, AUS; Funding acquisition: LCH, UB, MR, AUS; Supervision: MR and AUS; Writing—original draft: DYD, MR, AUS; Writing—review & editing: all authors.

## FUNDING INFORMATION

This work was supported by grants from the DFG (FerrOs‐FOR 5146) to LCH, UB, GS, MR, and AUS.

## DISCLOSURES

MR reports honoraria from UCB, Santhera, and Vifor for lectures and advisory boards. LH reports honoraria from Amgen, UCB, Ascendis, and Pharmacosmos for lectures and advisory boards. AUS reports a research grant from Pharmacosmos and from HemoClear, each for an investigator‐driven clinical trial. All other authors declare no conflicts of interest.

## Supporting information


Figure S1.

Figure S2.

Figure S3.

Text S1.

## Data Availability

The data that support the findings of this study are available on request from the corresponding author (MR).
