# Supplementary figures and images for: Bone phenotyping of murine hemochromatosis models with deficiencies of Hjv, Alk2, or Alk3: The influence of sex and the bone compartment
Source: FASEB J. 2024 Nov 15;38(22):e70179. doi: 10.1096/fj.202401015R (PMC11698015; doi:10.1096/fj.202401015R)

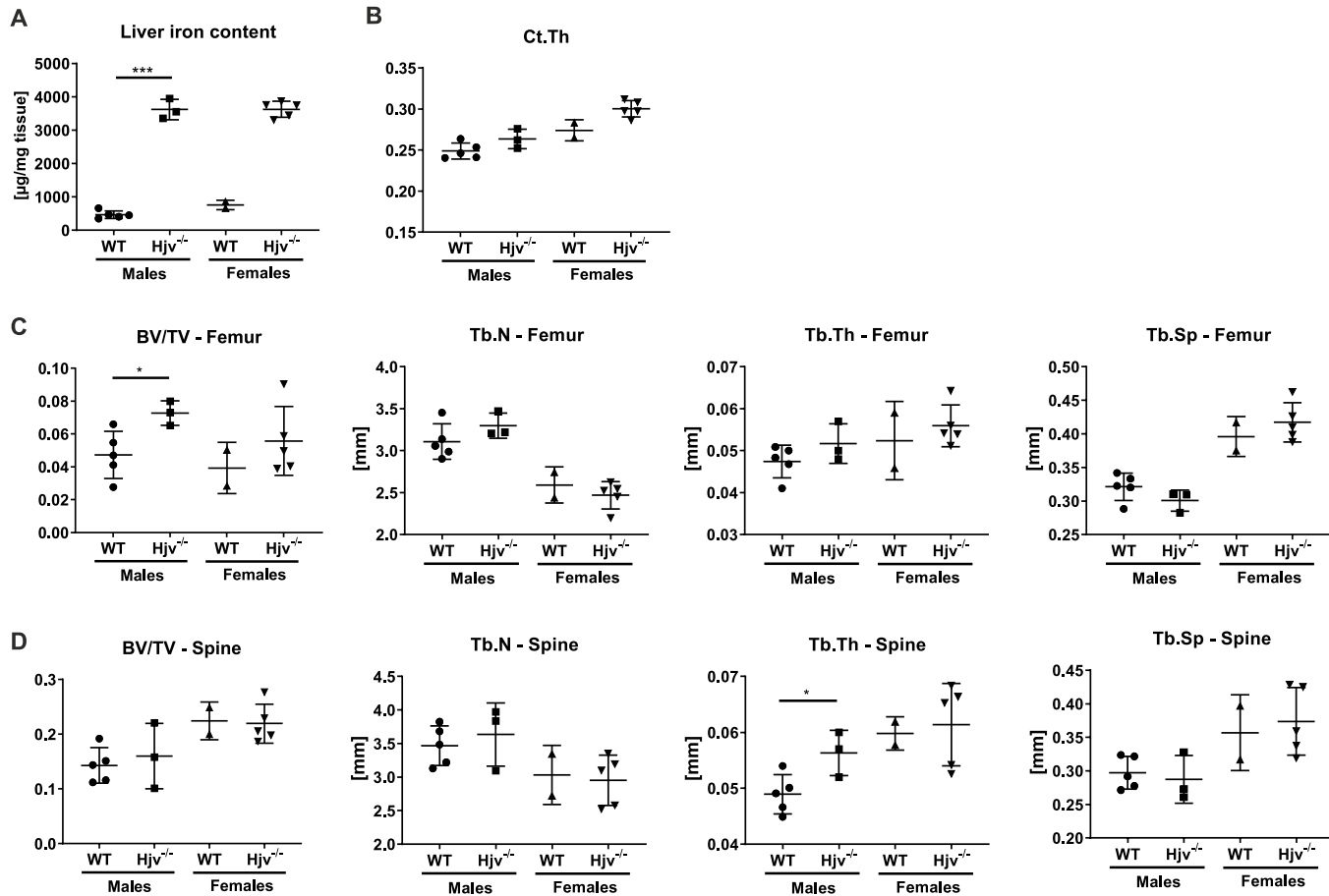

Supplement: Supplementary file 1 — Figure S1. [file FSB2-38-e70179-s004.pdf]

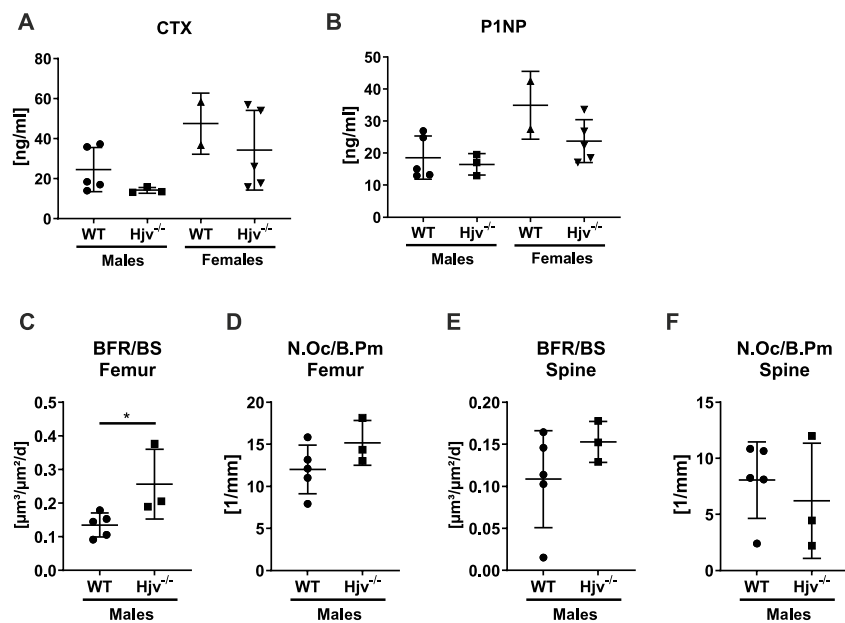

Suppl. Fig. 2

Supplement: Supplementary file 2 — Figure S2. [file FSB2-38-e70179-s001.pdf]

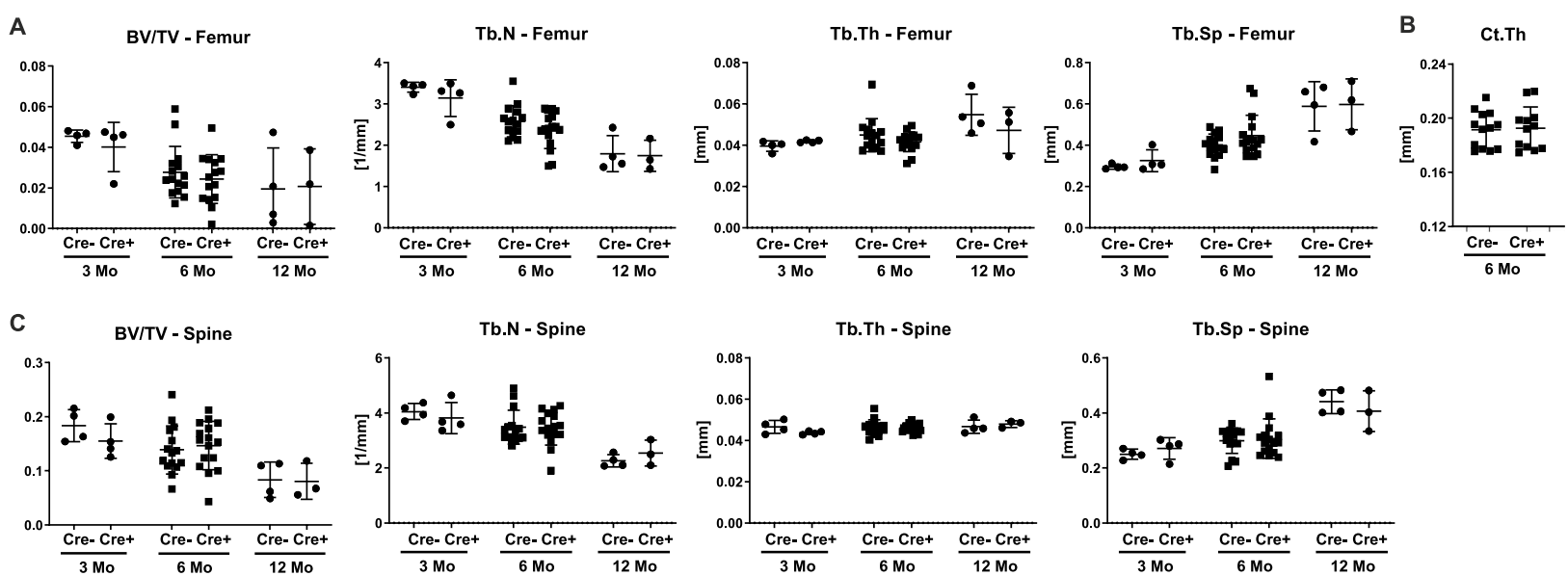

Supplement: Supplementary file 3 — Figure S3. [file FSB2-38-e70179-s003.pdf]
