# Supplementary material for: Bone phenotyping of murine hemochromatosis models with deficiencies of Hjv, Alk2, or Alk3: The influence of sex and the bone compartment
Source: FASEB J. 2024 Nov 15;38(22):e70179. doi: 10.1096/fj.202401015R (PMC11698015; doi:10.1096/fj.202401015R)
Supplement: Supplementary file 4 — Text S1. [file FSB2-38-e70179-s002.docx]

**Suppl. Figure 1. Bone phenotype of 12-month-old Hjv^-/-^ mice.** Twelve-months old male and female Hjv^-/-^ and wildtype mice were analyzed. **(A)** Liver iron content. **(B)** Cortical thickness (Ct.Th) of the femoral midshaft as measured with µCT. **(C)** Trabecular bone volume/tissue volume (BV/TV), trabecular number (Tb.N), thickness (Tb.Th) and separation (Tb.Sp) at the distal femur. **(D)** Trabecular bone volume/tissue volume (BV/TV), trabecular number (Tb.N), thickness (Tb.Th) and separation (Tb.Sp) at the fourth lumbar vertebrae. Individual dots represent individual mice. Mean and SD are indicated as horizontal lines. A two-sided *t*-test was used for statistical analysis. *p<0.05.

**Suppl. Figure 2. Serological and histological bone analyses of 12-month-old Hjv^-/-^ mice.** Twelve-months old male and female Hjv^-/-^ and wildtype mice were analyzed. **(A)** Serum C-terminal telopeptide of type I collagen (CTX) levels and **(B)** serum procollagen type I N-terminal peptide (P1NP) levels. **(C-F)** The bone formation rate per bone surface (BFR/BS) and the number of osteoclasts per bone perimeter (N.Oc/B.Pm) was assessed at the fifth lumbar vertebra and distal femur of male mice. Individual dots represent individual mice. Mean and SD are indicated as horizontal lines. A two-sided *t*-test was used for statistical analysis to compare genotypes of the same sex.

**Suppl. Figure 3. Bone phenotype of female Alk2^fl/fl^; Alb-Cre** **mice.** Bones of female Alk2^fl/fl^ controls and Alk2^fl/fl^; Alb-Cre mice of different ages were analyzed using µCT. **(A)** Trabecular bone volume/tissue volume (BV/TV), trabecular number (Tb.N), thickness (Tb.Th) and separation (Tb.Sp) at the distal femur, and **(B)** cortical thickenss at the femoral midshaft were analyzed using µCT. **(C)** Trabecular bone volume, trabecular number, thickness and separation at the fifth vertebral body. Indiviual dots represent individual mice. Mean and SD are indicated as horizontal lines. A two-sided *t*-test was used for statistical analysis to compare genotypes of the same age.
